# A Comprehensive Library of Familial Human Amyotrophic Lateral Sclerosis Induced Pluripotent Stem Cells

**DOI:** 10.1371/journal.pone.0118266

**Published:** 2015-03-11

**Authors:** Ying Li, Umamahesw Balasubramanian, Devon Cohen, Ping-Wu Zhang, Elizabeth Mosmiller, Rita Sattler, Nicholas J. Maragakis, Jeffrey D. Rothstein

**Affiliations:** 1 Department of Neurology, Johns Hopkins University, School of Medicine, Baltimore, Maryland, 21205, United States of America; 2 Brain Science Institute, Johns Hopkins University, Baltimore, Maryland, 21205, United States of America; 3 Department of Neuroscience, Johns Hopkins University, School of Medicine, Baltimore, Maryland, 21205, United States of America; 4 Ansari ALS Center for Stem Cell and Regeneration Research at Johns Hopkins, Baltimore, Maryland, 21205, United States of America; National Institute of Health, UNITED STATES

## Abstract

Amyotrophic lateral sclerosis is a progressive disease characterized by the loss of upper and lower motor neurons, leading to paralysis of voluntary muscles. About 10% of all ALS cases are familial (fALS), among which 15–20% are linked to Cu/Zn superoxide dismutase (SOD1) mutations, usually inherited in an autosomal dominant manner. To date only one FDA approved drug is available which increases survival moderately. Our understanding of ALS disease mechanisms is largely derived from rodent model studies, however due to the differences between rodents and humans, it is necessary to have humanized models for studies of disease pathogenesis as well as drug development. Therefore, we generated a comprehensive library of a total 22 of fALS patient-specific induced pluripotent stem cell (iPSC) lines. These cells were thoroughly characterized before being deposited into the library. The library of cells includes a variety of C9orf72 mutations, *sod1* mutations, FUS, ANG and FIG4 mutations. Certain mutations are represented with more than one line, which allows for studies of variable genetic backgrounds. In addition, these iPSCs can be successfully differentiated to astroglia, a cell type known to play a critical role in ALS disease progression. This library represents a comprehensive resource that can be used for ALS disease modeling and the development of novel therapeutics.

## Introduction

Amyotrophic lateral sclerosis (ALS), also known as Lou Gehrig’s disease, is a fatal disease characterized by the loss of upper and lower motor neurons, leading to paralysis of voluntary muscles [1]. The mechanisms involved in ALS pathogenesis are largely unknown [2]. About 10% of all cases are inherited, among which about 15–20% are linked to Cu/Zn superoxide dismutase (SOD1) mutations [3] and 40% to C9orf72 mutations [4,5]. Other genes, such as TDP-43, FUS/TLS [6], angiogenin [4,5,7], and very recently Matrin3 [8] have been also found to be linked to familial ALS (fALS). Insights from patient studies have been useful, but limited due to the inaccessibility of tissue from patients except postmortem specimens. While postmortem tissue can only provide end-stage changes, which are not typically suitable for mechanistic studies, other models are indispensible for ALS pathogenesis studies. One of the strategies is to generate rodent models with disease-specific mutations, such as different human SOD1 (hSOD1) mutations and TDP43 mutations. Some animals develop signs and pathological changes resembling those in patients [9–11], which are enormously valuable in disease study, however, not all transgenic mice with hSOD1 mutations develop the disease [12].

To date, only one drug, riluzole, is FDA approved for delaying disease progression for ALS patients with only modest efficacy in increasing survival [13]. The vast majority of novel therapeutics for ALS has advanced to the clinic following studies in rodent transgenic models of the mutant SOD1 form of ALS. Unfortunately, most drugs have failed Phase 2 and 3 trials, which can be due to several reasons, including (1) poor human and mouse trial design; (2) the mutant SOD1 mouse model may not be predictive of the pathophysiological process in the more common sporadic form(s) of ALS; (3) lack of proper pharmacokinetics, (4) lack of pharmacodynamic markers in human studies; (5) lack of evidence for target engagement by candidate drugs in human studies. In summary, it has been a growing concern that preclinical rodent models are not sufficiently predictive of complex neurodegenerative diseases [14].

Fortunately, significant progress in human induced pluripotent stem cell (iPSC) research provides a novel valuable tool for ALS research. Soon after the first reports on human iPSC generation [15,16], neurological disease specific iPSCs had been successfully generated from patients’ somatic cells [17–23], including several for ALS [18]. Remarkably, these cells can be differentiated to the type of cells which are critical for disease development, such as motor neurons from ALS-iPSCs [18,24–26], and they have been successfully used in disease modeling in neurological diseases like ALS, spinal muscular atrophy and familial dysautonomia [20,21,27]. ALS rodent studies have provided strong evidence that ALS is also a non-cell autonomous disease [28–32] as oligodendroglia may play a significant role in disease initiation and both astroglia and microglia play a role in disease progression. Further co-cultures of rodent glial cells, human fetal astrocytes overexpressing mutant hSOD1, or adult fALS and sALS astrocytes with motor neurons derived from human embryonic stem cells (hESCs) also showed non-cell autonomous effects on human motor neurons [27,33,34]. These studies together not only strongly suggest that non-cell autonomous mechanisms are involved in human ALS pathogenesis, but also provide evidence that patient specific iPSCs and hESCs are valuable for studying the disease. In addition, very recent studies with ALS C9orf72 iPS cell lines provide compelling evidence that the cells can have utility that exceeds prior disease models. These characteristics include: replication of actual human brain pathology, mimicking gene transcriptional changes seen in human disease, elucidating pathophysiological pathways and a cell culture platform for human drug screening and drug efficacy biomarkers[35][36].

Here we report that we generated a library of familial ALS patient-specific iPSCs (fALS-iPSCs) carrying a large variety of ALS mutations. These fALS-iPSCs can be differentiated to astroglia, a critical cell type that plays a role in ALS progression. In order to be widely useful, this “library” is based on the more common different ALS mutations, including SOD1 and FUS, and several lines of the same mutation are collected from different families. The fALS-iPSCs and their parental fibroblasts were generated to be publicly available to academic and commercial entities alike. The fibroblast lines have been reported separately [37]. We believe this widely available fALS-iPSC library will provide a great tool to study ALS pathophysiology, for biomarker development and for the preclinical development of novel therapies.

## Materials and Methods

### Patient Consent

The use of human tissues and associated demographic information was approved by the Johns Hopkins University Institutional Review Board and Ethics Committee (HIPAA Form 5 Exemption, Application #11–02–10–01RD). All skin biopsies were collected following written informed consent from the donor. The informed consent was approved by the Johns Hopkins University Institutional Review Board and Ethics Committee of Johns Hopkins Hospital (IRB protocol: NA-00021979). The consent allowed for use by all parties, commercial and academic, for research purposes only but not for human therapy.

### ALS-iPSC generation and characterization

The Sox2, Oct4, Klf4 and c-Myc encoding vectors were transduced into ALS-fibroblasts by using retrovirus based delivery. All iPS cell lines were created and initially characterized with an NIH-sponsored commercial agreement with iPierian (USA). Promising colonies were picked and evaluated for the expression of multiple pluripotent markers by quantitative PCR (qPCR) and/or immunocytochemistry. Their in vitro pluripotency was further determined by three germ layer differentiation via embryoid body formation. The transgene expression was determined by qPCR. Individual PCR reactions were normalized against *GAPDH* and plotted relative to the expression level in the control H9 and/or H7 Human ES cells. All iPS cell lines and fibroblasts were deposited with Coriell (http://www.coriell.org) for widespread public distribution.

### iPSC culture and differentiation

The iPSCs were maintained in mTeSR1 (StemCell Technology) and passed once a week using dispase (StemCell Technology) following the manufacturer’s instructions. Partially differentiated colonies were removed manually before differentiation analyses. The iPSCs were differentiated to neuroprogenitor cells (NPCs) via embryoid body (EB) formation by following the methods described previously [38]. To increase the efficiency of NPC differentiation, SMAD pathway was inhibited during neural induction, and the NPCs were then further differentiated to astrocyte by following the methods described previously [39,40].

### Immunocytochemistry

The cells were fixed with 4% paraformaldehyde at room temperature (RT) for 10min, and then permeabilized with 0.1% Triton X-100 in PBS at RT for another 10min. After incubation with 10% donkey serum or 3% BSA for 20min at room temperature, the primary antibody was applied and incubated at RT for 1hr and Alexa Flour 488 or 555 labeled secondary antibody for another hour. Nuclei were stained with DAPI. Images were randomly taken using a Nikon microscope with SPOT camera. Primary antibodies used in the study are listed in S1 Table.

### Statistical analysis

Statistic analysis was performed by Prism (GraphPad Software). Student’s t test was used for the percentage of rosette positive colony comparison between control and SOD1 groups. One-way ANOVA with Tukey’s post analysis was carried out to compare maker expression at different time points. P<0.05 was considered significant. Data are summarized from more than three independent experiments and shown as mean±SEM.

## Results

### Generation and characterization of fALS-iPSCs

**Fig 1:**
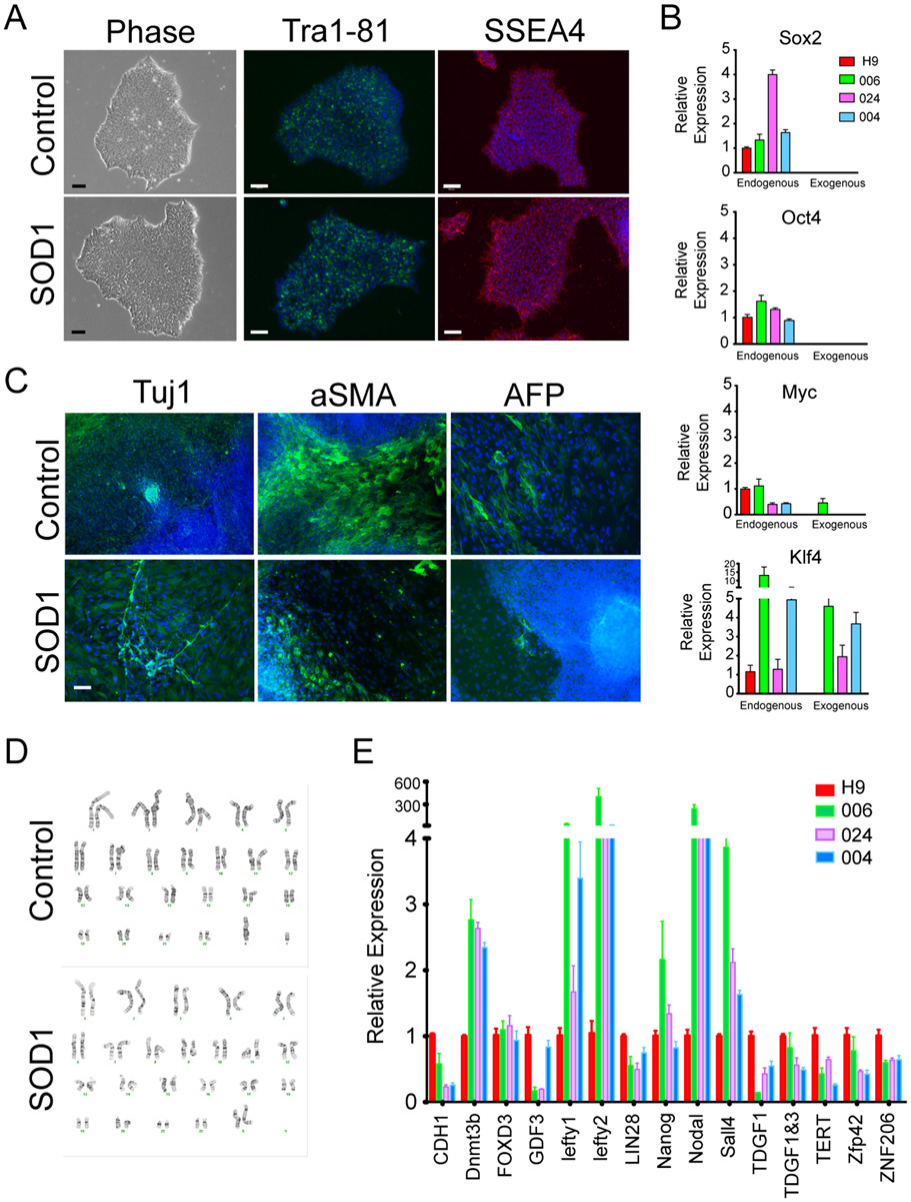
Characterization of fALS-iPSCs. (A) Representative pictures of control- (006) and SOD1-iPSC (002) morphology and their expression of Tra1–81 (green) and SSEA4 (red). Nuclei were stained with DAPI (blue). Size bar: 100μm. (B) Representative qPCR evaluation of endogenous and transgene expression by control- (006) and SOD1-iPSCs (004 and 024). (C) Representative pictures show both control (006) and SOD1-iPSCs (024) generated cells representing the three embryonic germ layers (Tuj1, aSMA and AFP, all in green). Nuclei were stained with DAPI (blue). Size bar: 100μm. (D) Representative karyotypes of control- (006) and SOD1-iPSCs (024). (E) Representative qPCR results of pluripotent marker expression by control (006) and SOD1-iPS (004 and 024) lines.

**Fig 2:**
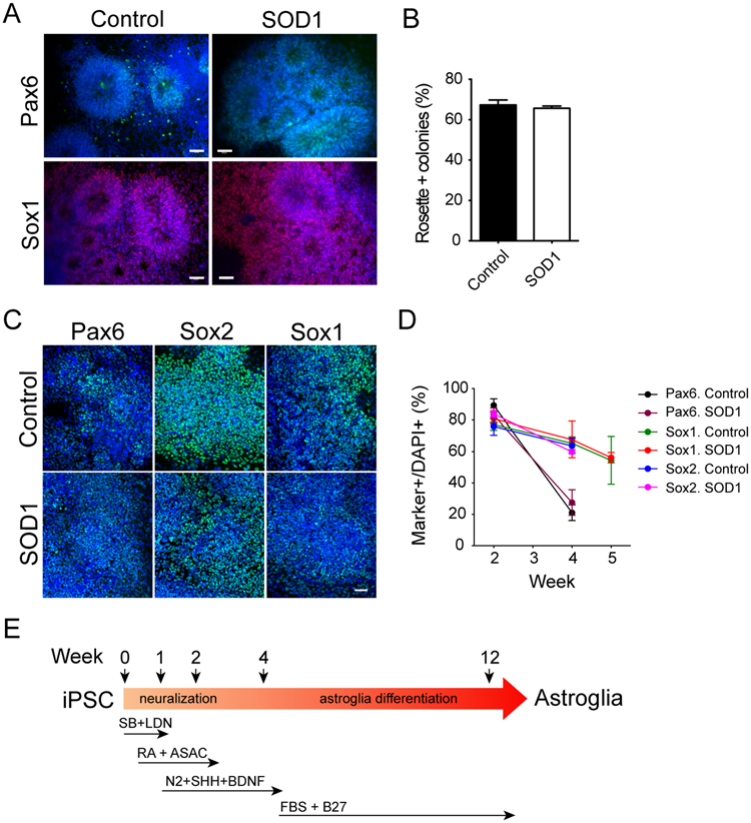
Differentiation of SOD1-iPSCs to NPCs. (A, B) NPCs were generated via EB formation. (A) Rosettes and neural tube structures were formed and the cells expressed Pax6 and Sox1. Representative pictures were taken from 006 control and 002 SOD1 lines. Nuclei were stained with DAPI. (B) Quantification of colonies with rosette structures. (C, D) NPCs were induced by inhibition of SMAD pathway. Most cells expressed Pax6, Sox2 and Sox1 at week 2. Nuclei were stained with DAPI. (D) Dynamic examination of Pax6, Sox1 and Sox2 expression by NPCs after 2–5 week. (E) Time line of neural induction by the inhibition of SMAD pathway and astrocyte differentiation. Size bar, 50μm.

The human (healthy and ALS subject) dermal fibroblasts were collected through a nationwide National Institutes of Health supported collaboration (Johns Hopkins University School of Medicine, Massachusetts General Hospital and Emory University ALS Center). The fibroblast cultures were established at Johns Hopkins School of Medicine for iPSC generation. A total of four healthy control iPSC lines, twenty-two fALS-iPSC lines and two sporadic ALS-iPSC lines with c9of72 repeat mutation were generated (summarized in Table 1). These mutations cover twelve different *sod1* mutations, three *FUS* mutations, one angiogenin (*ANG*) and one *FIG4* mutation. All iPSCs have been generated by using Sox2, Oct4, Klf4 and c-Myc factors delivered by retroviruses with a programming efficiency of ~0.1%. Putative iPSCs underwent a validation profile that included: 1) morphological analysis, compact colonies with clear boundary and are comprised of cells with big nucleus and large nucleoli and scant cytoplasm (Fig. 1A); 2) pluripotent gene expression by quantitative PCR, including CDH1, Dnmt3b, FoxD3, GDF3, Lefty1/2, LIN28, Nanog, Nodal, Sall4, TDGF1, TDGF1&3, TERT, Zfp42 and ZNF206, and their expression were comparable with hESCs (H9 and/or H7) (Fig. 1E and S1 Fig.); 3) pluripotent marker expression by immunocytochemistry, such as Tra1–81 and SSEA4 (Fig. 1A), Tra1–60 and SSEA3 (not shown), 3) silencing of transgenes and upregulation of endogenous Sox2, Oct4, Klf4 and Myc (Fig. 1B and S1 Fig.); 4) in vitro differentiation to three germ layers via EB formation (Fig. 1C and S2 Fig.). The expression of ectoderm cell markers such as neuron marker Tuj1, mesoderm cell markers such as smooth muscle antigen (aSMA) or desmin, and endoderm cell markers alpha fetoprotein (AFP) or forkhead box protein A2 (FoxA2), were evaluated by immunocytochemistry; 5) normal karyotypes (Fig. 1D and S3 Fig.). Only cells meeting all these criteria were deposited into the library. The cells were maintained in mTeSR1 medium.

**Table 1:** fALS-iPSC lines.

ID	Gene	Mutation	Age at biopsy (year)	Disease length at Biopsy (month)	Clinical site at onset	Gender	Race
**Control**							
**005**	Control	NA	66	NA	NA	M	W
**006**	Control	NA	68	NA	NA	M	W
**010**	Control	NA	50	NA	NA	F	W
**018**	Control	NA	55	NA	NA	F	W
**Familial ALS**							
**002**	SOD1	A4V	57	NA	NA	F	W
**007**	SOD1	A4V	40	NA	NA	F	W
**008**	SOD1	A4V	45	NA	NA	F	W
**013**	SOD1	A4V	63	10	spinal	F	W
**021**	SOD1	C38G	47	34	spinal	M	A
**004**	SOD1	D90A	50	96	spinal	F	W
**028**	SOD1	D90A	68	83	spinal	F	W
**017**	SOD1	D91A	56	103	spinal	M	W
**026**	SOD1	E100G	41	47	spinal	M	W
**024**	SOD1	E49K	49	36	spinal	F	W
**033**	SOD1	G86R	77	NA	NA	M	W
**003**	SOD1	I112T	44	NA	NA	F	W
**016**	SOD1	I113T	53	60	spinal	M	W
**015**	SOD1	L144P	51	30	spinal	M	W
**001**	SOD1	N139K	46	46	spinal	M	AA
**009**	SOD1	V148G	54	6	spinal	M	W
**027**	ANG	ND	55	10	bulbar	M	W
**014**	FIG4	ND	67	12	bulbar	F	W
**025**	FUS	H517Q	50	96	spinal	F	AA
**031**	FUS	T198C	57	23	bulbar	M	W
**023**	FUS	G522A	37	13	spinal	M	W
**034**	VCP	ND	64	132	spinal	F	W
**C9orf72**							
**033**	C9orf72	>800 repeats	62	43	Spinal	M	W
**034M**	C9orf72	>800 repeats	65	33	Bulbar	F	W

Note: NA, not applicable/not known; ND, not determined; M, male; F, female; W, white; AA, African American.

### Differentiation of fALS-iPSCs to NPCs

ALS-iPSCs have been successfully differentiated to motor neurons [18], but there were no detailed reports whether the presence of mutant SOD1 interferes with neural progenitor differentiation. In the differentiation studies, we focused on iPSC lines with *sod1* mutations (line 001, 002, 003, 008, and 013), and controls (line 005, 006 and 018). We first followed the well-established method to differentiate the cells to NPCs via EB formation. Both control and SOD1-iPSC generated rosettes structure after about ten days of induction and developed to neural tube like rosettes at around 12–14 days. The cells in the neural tube like structures expressed NPC marker Pax6 and Sox1 (Fig. 2A), and no significant differentiation differences were seen between control and SOD1-iPSCs (control 67.3±2.4% vs 65.6±1.2%, Fig. 2B), suggesting that the presence of mutant SOD1 had no obvious effects on neural induction. As inhibition of SMAD pathway can increase the efficiency of neural induction [39,40], we next applied SB431542 and LDN193189 for one week for neutralization following the protocol developed by our ALS consortium [40]. Both control and SOD1-iPSCs were efficiently differentiated to NPCs expressing Pax6 (control 89.3±4.2% vs SOD1 83.1±4.5%, P>0.05), Sox1 (control 77.2±3.7% vs SOD1 80.8±3%, P>0.05) and Sox2 (control 76.1±5.6% vs SOD1 83.9±2.1%, P>0.05) at week 2 of induction (Fig. 2C). The expression of Pax6 dramatically decreased at week 4 (control 21±5% vs SOD1 27.6±8.2%, P>0.05), and Sox1 (control 65.4±4.1% vs SOD1 67.7±11.6% at week 4, P>0.05; control 54.5±15.3% vs SOD1 56.2±3.3% at week 5, P>0.05) and sox2 (control 63.7±4.9% vs SOD1 59.9±1.4%, P>0.05) gradually decreased at week 4–5 (Fig. 2D) when the medium was changed for further astroglia differentiation. No significant differences in each marker expression between control and SOD1-iPSCs were observed at each time point. Similar differentiation was seen with the C9orf72 iPS cell lines (non shown). Notably for the C9orf72 iPS cell lines previously we demonstrated that the expansion mutation was maintained [35].

### Differentiation of fALS-iPSCs to astroglia

**Fig 3:**
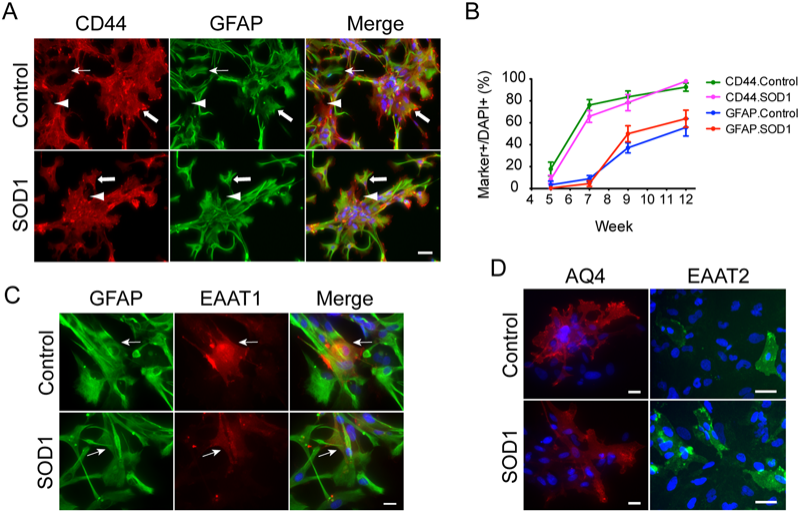
Differentiation of SOD1-iPSCs to astroglia. Representative pictures of control (006) and SOD1-iPSC (008) derived astrocyte after 15-week differentiation showing astroglial marker expression. (A) CD44 (red) and GFAP (green) expression by differentiated cells. Thin arrows indicate GFAP+ only cells. Arrowheads indicate CD44+ only cells. Thick arrows indicate CD44+/GFAP+ cells. (B) Quantification of CD44+ and GFAP+ cells at different time points. (C) EAAT1 (red) expression by GFAP+ (green) astrocytes. Arrows indicate double positive cells. (D) Aquaporin 4 (AQ4, red) and EAAT2 (green) expression. Nuclei were stained with DAPI (blue). Size bar, 20μm.

To test whether the iPS cells in this library can be efficiently differentiated to astroglia and whether these allelic mutations could alter that process, we followed the protocol developed by our ALS consortium [40]. We treated the NPCs, which were induced by inhibition of SMAD pathway, with fetal bovine serum (FBS) to induce astrogliogenesis. The NPCs changed their morphology dramatically to a more flattened shape after about two weeks. Both control and SOD1-iPSCs expressed the astroglia progenitor marker CD44 [41] as early as 5 weeks in a low percentage of cells (control 17.8±6.3% vs SOD1 8.3±3.7%) and rapidly increased over the next two weeks (control 76.2±5% vs SOD1 65.8±5.4% at week 7) followed by steady increases (from week 9 to week 11, control 83.6± 5.4% to 92.6± 3.7% vs SOD1 78.4± 7.3% to 97.9± 0.9%) (Fig. 3C). GFAP expression was observed as early as week 4, but the cells were bipolar and had long processes, which we previously interpreted as radial glial and not bona fide astroglia [40]. Cells with flattened morphology started to express GFAP as early as week 5 but at very low percentage (control 3.4±3.4% vs SOD1 0.4±0.1%). In the next four weeks GFAP expression increased rapidly (from week 7 to 9, control 9±2.9% to 37.2±4.6% vs SOD1 4.5±2.6% to 50±7.3%), and then increased steadily (week 12–13, control 55.8±7.9% vs SOD1 63.8±7.8%) (Fig. 3B). After 7 weeks, most cells showed flattened morphology, and most of them expressed CD44. CD44+/GFAP-, CD44+/GFAP+ and CD44-/GFAP+ cells were easily observed after week 9, suggesting astroglia progenitors differentiated toward astroglia steadily, but slowly. After 13 weeks, a very small percentage (less than 10%) of cells expressed more mature physiologically and synaptically relevant astroglial markers, EAAT2 (known as GLT1 in rodents), EAAT1 (known as GLAST in rodents), and aquaporin 4 (Fig. 3A), suggesting that the cells were developing appropriate astroglial functional properties. We did not observe significant differences in these maturation markers between control and mutant SOD1 expressing cells at any analyzed time points. The small percentages of cells expressing the mature astroglial marker EAAT2, for example, suggests that, while these cells have mature features of astroglia, they likely remain immature astrocytes at the in vitro time points studied, as suggested by the previous study in a very small number of iPS lines [40].

During the differentiation, we also observed that most of the differentiating cells started to express S100beta and vimentin as early as week 4–5 and reached peak (near 100%) at week 5–6 (not shown). We further examined the cells for any evidence of mutation altering biology. None of the GFAP+ astroglia with SOD1 mutations had evidence of aberrant morphology compared to the control cells. In addition, no evidence for intracellular, either cytoplasmic or nuclear, inclusions with ubiquitin immunoreactivity has been seen (not shown).

## Discussion

In 2009, through the American Recovery and Reinvestment Act (ARRA), the National Institutes of Health (NINDS) funded three consortia to develop well-characterized and publicly available iPSCs from familial forms of ALS, Parkinson’s disease (PD) and Huntington’s disease (HD). As a member of this consortium, we were tasked with generating a comprehensive library of fALS-iPSCs, which includes 22 fALS-iPSC and 4 control lines. This consortium was assembled to collect fALS biopsies and fibroblasts, including relevant clinical information, generating initial iPSC lines, employing a commercial collaborator (iPierian) specialized in rapid reliable development of iPSC lines, and validating their basal biology to differentiate into astroglia and motor neurons. In this report we outline this large collection of diverse fALS-iPSC lines and their reliable differentiation to astroglia. Although sporadic ALS iPS cell lines could be useful as well, early review of the program provided support for only the generation of cell lines from available fALS patients. Subsequently, our labs have collected over 100 fibroblast lines from sporadic ALS as well (unpublished). Most recently, we generated several c9orf72 ALS/FTD iPS cell lines from sporadic ALS patients (see above) [42].

iPSCs can be generated from different tissue resources [43–45] by using virus-based delivery of transcription factors [16,46–48], direct delivery of DNA [49–52] or modified mRNA transcripts [53], small molecules or proteins [54]. The cells in the present library have been generated by using Yamanaka’s four factors, which was the more mature methodology when the library was being generated and gave better reprogramming efficiency. Recently, other approaches have been employed focused on generating “print free” cell lines (without exogenous gene insertion or retention in the chromosome) and these approaches either suffer from poor efficiency or are costly and time-consuming. As cells generated by different laboratories may have their laboratory-specific molecular and/or genetic “finger print” [55], it could prove difficult to make comparisons between analyses using the cells generated from different resources. All the ALS-iPSCs in this library have been generated in collaboration with iPierian using the same reprogramming methodology, chosen using the same quantitative control, and cultured under the same conditions, which provided maximum control of cell qualities.

We were able to successfully differentiate fALS-iPSCs with different mutant *sod1* primarily into rudimentary GFAP expressing-astroglia and some biologically relevant mature astroglia expressing EAAT1, EAAT2 or aquaporin 4. In the previous report, hESC and healthy human iPSC lines were differentiated to functional astroglia [40]. By using the same protocol, we observed comparable efficiency of NPC and astroglia differentiation of the fALS-iPSCs from this library in terms of the expression of NPC marker Pax6, astroglia progenitor markers CD44 and mature astroglia markers EAAT2, EAAT1 when induced by low percentage of FBS. Therefore, it is very possible the iPSCs in this library can be differentiated to mature functional astroglia by treating with FGF1 or FGF2 as shown in the previous study. However, whether the presence of mutant SOD1, or other ALS mutations, would interfere with astroglia maturation needs further study.

The astroglia derived from this fALS-iPSC library represent the natural disease in terms of allelic copies of the mutant gene, which would provide a faithful model to study cell-cell interactions responsible for disease pathophysiology in ALS and astroglia pathophysiology. From the mutations we tested, there was no gross evidence of aberrant cellular morphology or intracellular inclusions under basal conditions. It is possible that the majority of the cells were immature astroglia, or the cells were not purified or enriched, or extrinsic signals (such as from neurons) are necessary. In vivo studies from both human and mice showed there is dramatic dysregulation of EAAT2 expression in disease and EAAT2 expression is tightly coupled to the presence of neurons [56], future studies could examine the dysregulation of the human iPSC derived astroglia in human neuron-astroglia co-cultures.

In addition to its application in disease modeling, the fALS-iPS derived cells offer a valuable tool for screening and validating CNS compounds. Candidate compounds for treating CNS defects fail in clinical trials in over 90% of cases due to poor targeting, lack of efficacy, and unacceptable side effects [57]. Recent studies using ALS motor neurons derived from iPSCs with mutant TDP43 or SOD1 were successfully used in drug screening [24,58], supporting the use of iPSC cell culture platforms for drug discovery efforts. Similarly, we have used ALS iPS neurons derived from C9orf72 mutant patients to successfully generate and validate antisense oligonucleotide candidate therapies [42]. The present library covers 12 *sod1* mutations of the known >105 *sod1* mutations [59], including A4V mutation, the most prevalent mutation in the US which causes rapidly progressing ALS, and D90A mutation that is correlated with slowly progressing ALS. These different mutations show different enzyme activities and/or thermal stabilities. Importantly, there is more than one line available from certain mutations, such as A4V and D90A. Given that these cells were from different families, this could provide important insights into the variability of background genetics, which can influence the properties of the lines and their utility for drug screening. Therefore, this library provides a very valuable resource for drug screening, to test drug toxicity and efficacy among subpopulations [60] as so called “in vitro clinical trial” [61].

During astroglia differentiation, most cells expressed markers of astroglial progenitor fate compared to GFAP and mature astroglial markers. The generation and maturation of iPS derived-astroglia takes a long time in vitro, which is consistent with human embryonic astroglial development. However, this lengthy time to differentiate provides a platform to screen drugs which may facilitate astroglia development and maturation. For example, as EAAT2 expression is downregulated in ALS [62], maintenance and up-regulation of EAAT2 expression in astrocytes promotes motor neuron survival and prolongs disease duration [63,64]. Screening and validating drugs for EAAT2 expression using ALS-iPSC derived astroglia can potentially provide a therapy for ALS patients.

## Supporting Information

S1 Fig qPCR analysis of pluripotent stem cell marker and transgene expression by fALS-iPS cells. TaqMan assay was used to determine gene expression. The log ratio is the difference in Ct between gene of interest and the housekeeping gene GAPDH.
(PDF)

S2 Fig In vitro differentiation potential of fALS-iPS cells. Embryoid Body culture was used to examine the differentiation potential of the iPSC lines. These aggregates are allowed to grow for several days and then assessed by antibody staining for their ability to differentiate into cell types representing three germ layers.
(PDF)

S3 Fig Karyotypes of fALS-iPS cells. The iPS cells were analyzed between passage number 4 to 10 (P4—P10). Line 010 showed balanced translocation due to reciprocal exchange between the long-arm of chromosome 1 and the short-arm of chromosome 17.
(PDF)

S1 Table Antibodies used in the study. (DOCX)
