# Supplementary figures and images for: A Comprehensive Library of Familial Human Amyotrophic Lateral Sclerosis Induced Pluripotent Stem Cells
Source: PLoS One. 2015 Mar 11;10(3):e0118266. doi: 10.1371/journal.pone.0118266 (PMC4356618; doi:10.1371/journal.pone.0118266)

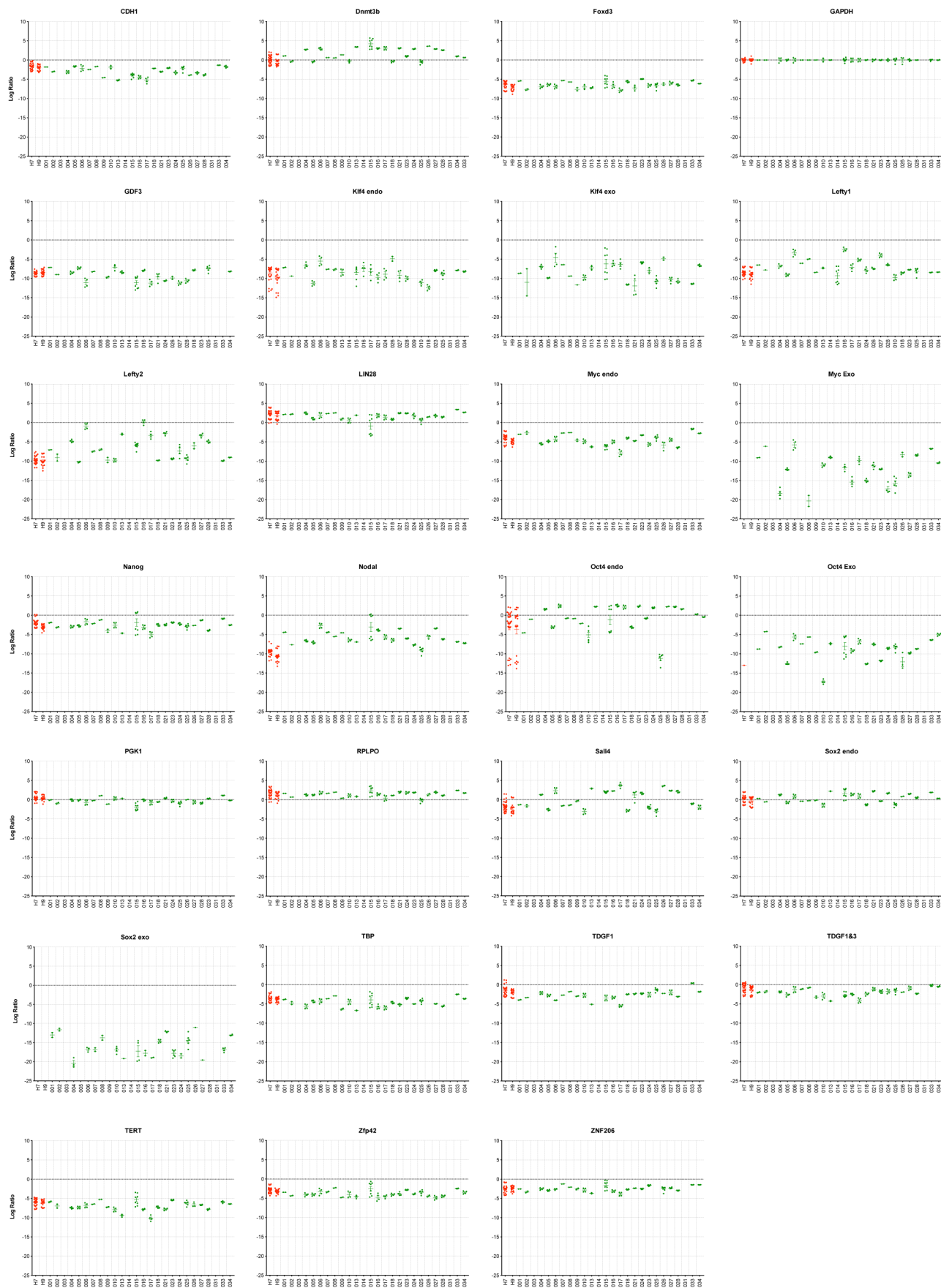

Supplement: S1 Fig — TaqMan assay was used to determine gene expression. The log ratio is the difference in Ct between gene of interest and the housekeeping gene GAPDH. (PDF) [file pone.0118266.s001.pdf]

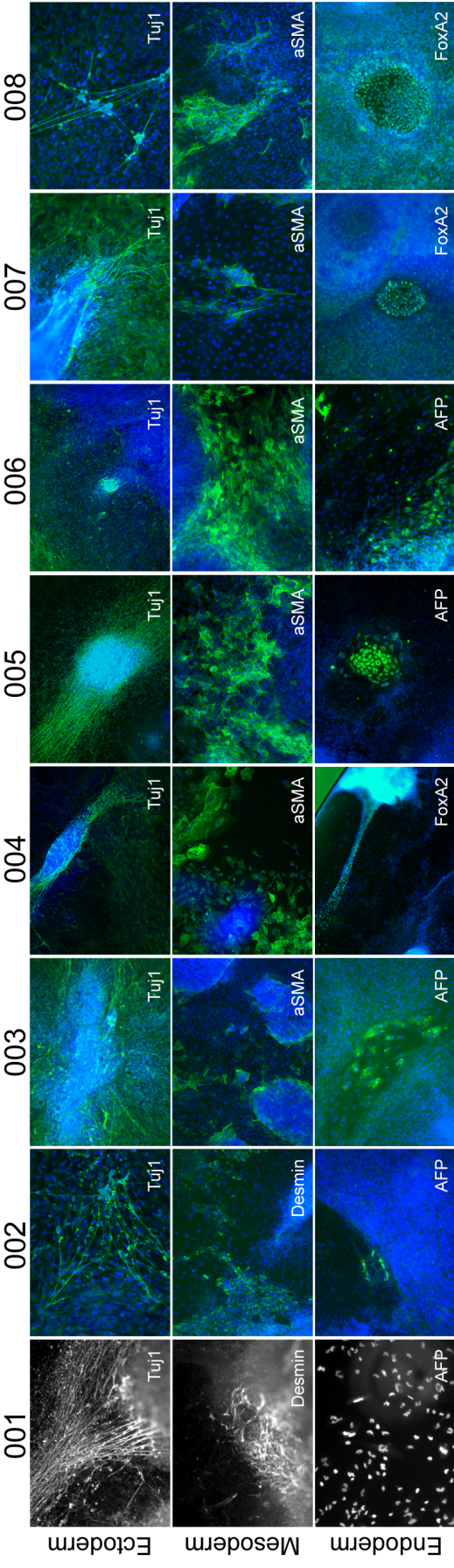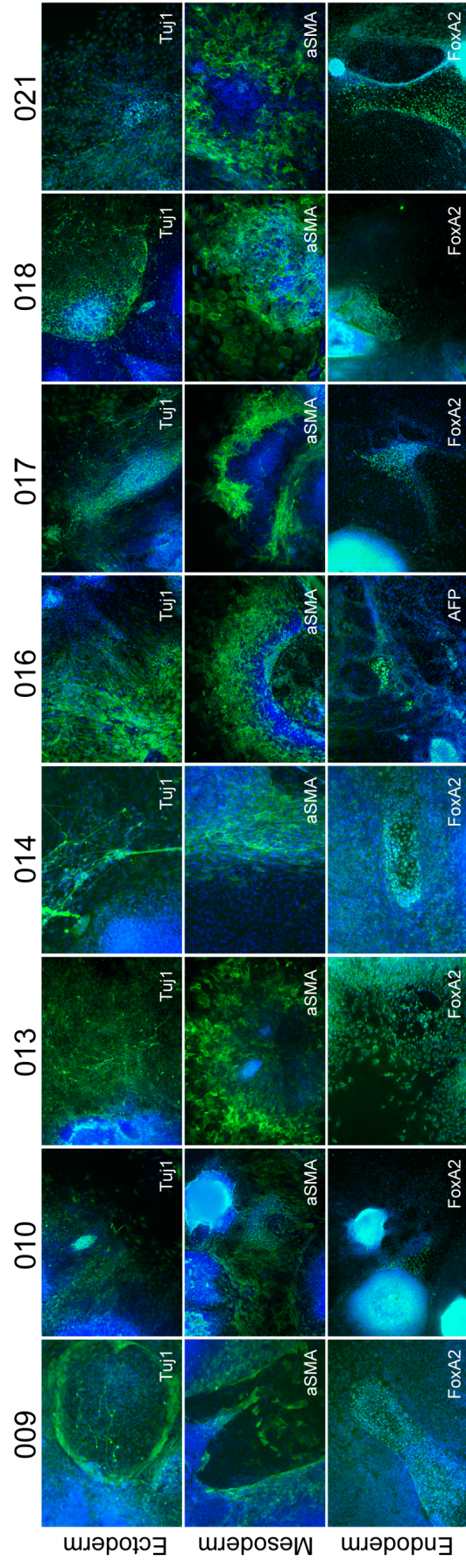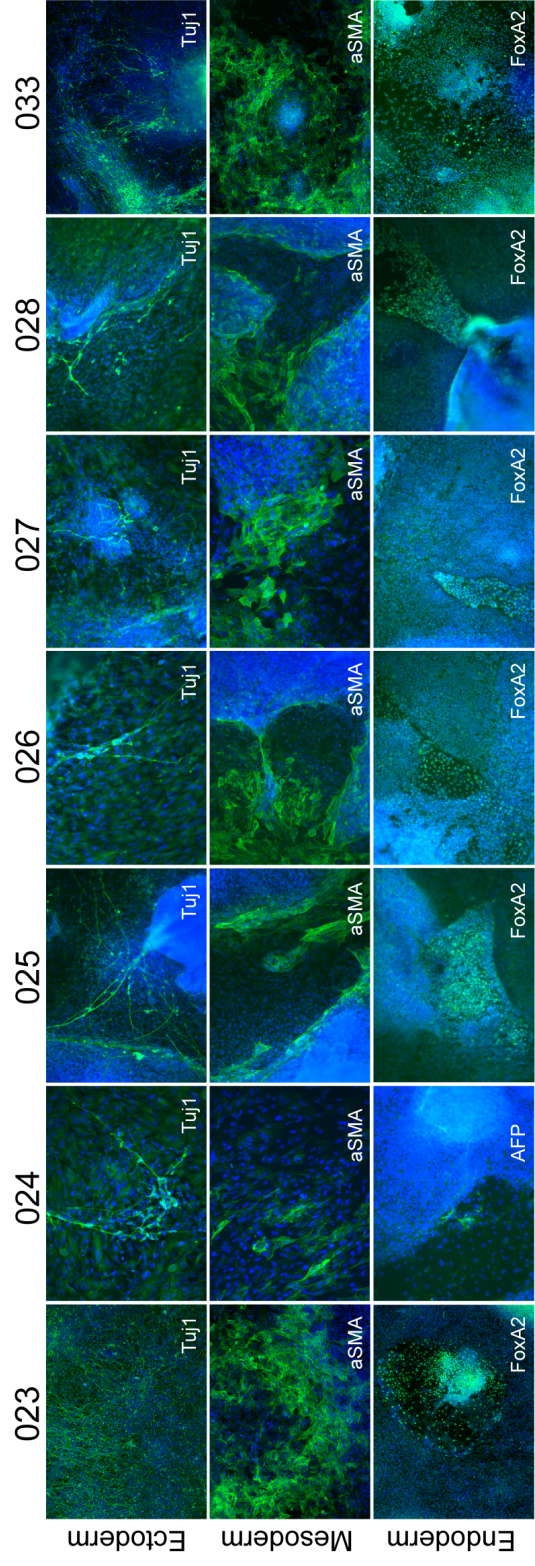

Supplement: S2 Fig — Embryoid Body culture was used to examine the differentiation potential of the iPSC lines. These aggregates are allowed to grow for several days and then assessed by antibody staining for their ability to differentiate into cell types representing three germ layers. (PDF) [file pone.0118266.s002.pdf]
