# Supplementary material for: A Comprehensive Library of Familial Human Amyotrophic Lateral Sclerosis Induced Pluripotent Stem Cells
Source: PLoS One. 2015 Mar 11;10(3):e0118266. doi: 10.1371/journal.pone.0118266 (PMC4356618; doi:10.1371/journal.pone.0118266)
Supplement: S3 Fig — The iPS cells were analyzed between passage number 4 to 10 (P4—P10). Line 010 showed balanced translocation due to reciprocal exchange between the long-arm of chromosome 1 and the short-arm of chromosome 17. (PDF) [file pone.0118266.s003.pdf]

001

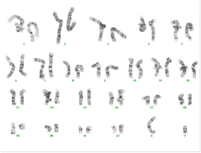

P10
46, XY[20]

002

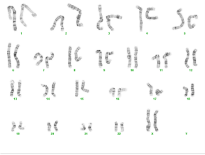

P4
46,XX[20]

003

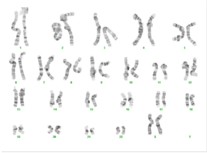

P5
46, XX[20]

004

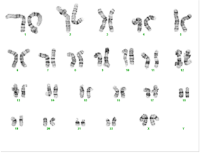

P4
46, XX[20]

005

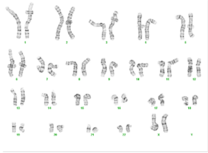

P7
46, XX[20]

006

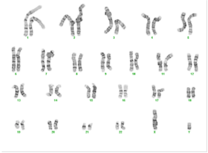

P7
46,XY[20]

007

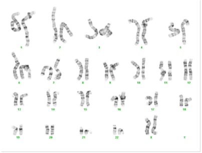

P5
46, XX[19]

008

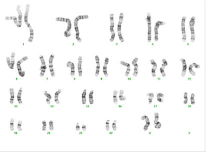

P5
46, XX[20]

009

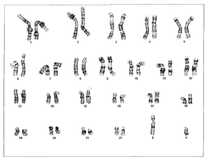

P5
46, XY[17]

010

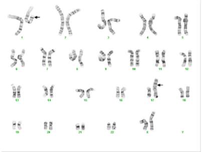

P8
46, XX,t(1:17)(q32;p13)[20]

013

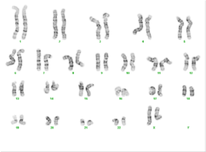

P4
46, XX[20]

014

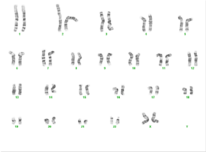

P6
46, XX[18]

015

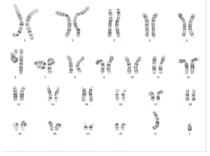

P5
46, XY[19]

016

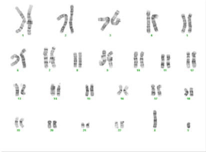

P5
46, XY[19]

017

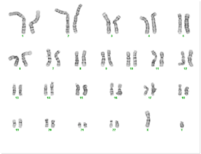

P6
46, XY[14]

018

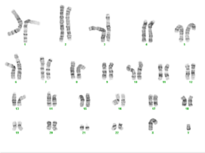

P6
46, XY[19]

021

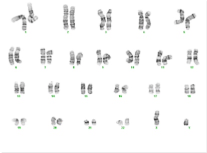

P5
46, XY[19]

023

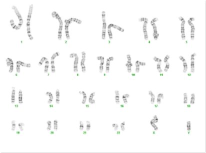

P6
46, XY[19]

024

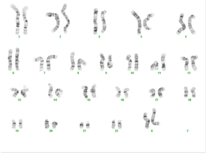

P4
46, XX[20]

025

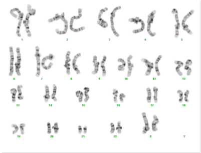

P4
46, XX[20]

026

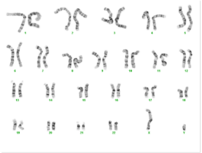

P6
46, XY[20]

027

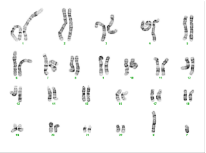

P6
46, XY[20]

028

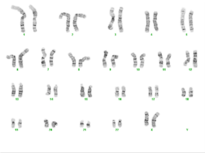

P6
46, XX[20]

031

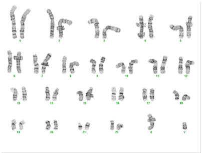

P6
46, XY[17]

033

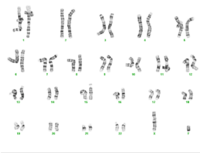

P9
46, XY[20]

034

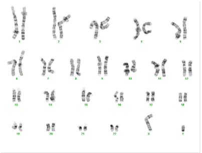

P6
46, XY[20]
