# Supplementary material for: A Comprehensive Library of Familial Human Amyotrophic Lateral Sclerosis Induced Pluripotent Stem Cells
Source: PLoS One. 2015 Mar 11;10(3):e0118266. doi: 10.1371/journal.pone.0118266 (PMC4356618; doi:10.1371/journal.pone.0118266)
Supplement: S1 Table — (DOCX) [file pone.0118266.s004.docx]

Supplementary table 1. Antibodies used in the study

|  | Species | Dilution | Sources |
| --- | --- | --- | --- |
| Aquaporin 4 | Rabbit | 1:200 | Sigma |
| CD44 | Rat | 1:100 | BD Pharmingen |
| GFAP | Rabbit | 1:1000 | DAKO |
| EAAT1 (GLAST) | Mouse | 1:50 | Miltenyi Biotech |
| EAAT2 (GLT1) | Rabbit | 1:500 | Rothstein lab |
| Iba1 | Rabbit | 1:1000 | WAKO |
| Nestin | Rabbit | 1:400 | Millipore |
| Pax6 | Mouse | 1:5000 | DSHB |
| S100beta | Mouse | 1:250 | Sigma |
| Sox1 | Goat | 1:500 | R&D |
| Sox2 | Mouse | 1:400 | R&D |
| SSEA3 | Rat | 1:100 | Millipore |
| SSEA4 | Mouse | 1:100 | Millipore |
| Tra1-60 | Mouse | 1:100 | Millipore |
| Tra1-81 | Mouse | 1:100 | Millipore |
| Ubiquitin | Rabbit | 1:200 | DAKO |
| Vimentin | Mouse | 1:300 | Sigma |
